# Wind tunnel system to apply precooling airflow and cooling conditions for fruit chilling injury assessment^☆^^☆☆^

**DOI:** 10.1016/j.mex.2026.103813

**Published:** 2026-02-03

**Authors:** Eugene Sadie, Corne Coetzee, Mike Owen, Marli Kleyn, Tarl Berry

**Affiliations:** a Department of Mechanical and Mechatronic Engineering, Stellenbosch University, Stellenbosch, South Africa; b Citrus Research International, Department of Horticultural Sciences, Stellenbosch University, Stellenbosch 7600, South Africa; c Africa Institute for Postharvest Technology, Department of Horticultural Sciences, Faculty of AgriSciences, Stellenbosch University, Stellenbosch 7600, South Africa

**Keywords:** Forced-air cooling, Static cooling, Precooling, Postharvest cooling, Citrus, Fruit, Chilling injury

## Abstract

Chilling injury (CI) affects up to 33% of globally traded postharvest commodities, yet the influence of precooling conditions on CI susceptibility remains largely unexplored. This is likely due to the inherent complexity and variability of commercial operations. This study introduces a novel methodology to systematically evaluate precooling-CI interactions under controlled laboratory conditions. The approach is grounded in commercial precooling characteristics and comprehensive cold chain CI evaluation protocols. A laboratory forced-air cooling system was developed, consisting of wind tunnels installed in existing cold rooms to replicate commercial precooling airflow speeds and cooling rates. The wind tunnels operate over a 0.0–0.9 m s^-1^ range, with an uncertainty of <3.6% at a 99% confidence level. The system successfully reproduced commercial precooling conditions and induced statistically significant, differentiable CI responses in citrus trials. The methodology provides researchers with a reproducible platform for investigating precooling optimisation strategies and CI mitigation for both citrus and other fruit types, with potential applications in postharvest technology development and supply chain optimisation.

Key methodological components include:

• Characterisation of commercial precooling conditions to establish realistic design parameters
• Development and application of a laboratory-scale wind tunnel precooling simulator
• Chilling injury evaluation protocol for precooling protocol assessment

## Graphical abstract

**Figure:**
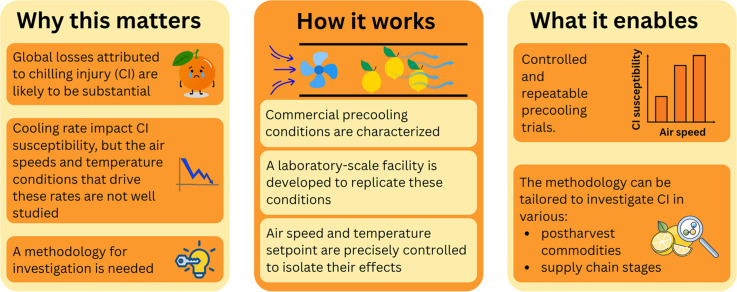

**Specifications table**

**Table:** 

Subject area	Engineering
More specific subject area	Forced convection precooling of fresh produce
Name of your method	Method to develop a laboratory forced air cooling system
Name and reference of original method	N/A
Resource availability	https://doi.org/10.5281/zenodo.15726691

**List of symbols**

**Table:** 

**Symbol**	**Description**	**Unit**
$CI_{index}$	Chilling injury index	—
$CI_{induced}$	Total percentage of fruit with CI	%
$CI_{severity}$	Chilling injury severity (0–3)	—
ΔP	Pressure differential across pallet	Pa
c	Regression offset	—
l	Produce depth relative to airflow direction	m
m	Regression gradient	—
n	Number of sampling intervals	—
$N_{score,i}$	Number of fruit in each score class	—
$N_{affected}$	Number of fruit with CI	—
$N_{evaluated}$	Total number of fruit evaluated	—
$\sigma_{calibration}$	Calibration uncertainty	m s⁻¹
$\sigma_{max}$	Maximum deviation from setpoint	m s⁻¹
$\sigma_{std}$	Standard deviation of air speed	m s⁻¹
$\sigma_{total}$	Total uncertainty	m s⁻¹
$\bar{u}$	Superficial inflow air speed	m s⁻¹
ω	Directional airflow resistance coefficient	Kg m⁻⁴
Z	Stability metric (confidence interval ratio)	—

## Background

Postharvest fruit and vegetable commodities account for 23 % of global trade, representing a multi-billion-dollar (US) industry [1]. However, this economic potential is constrained by the short shelf life and significant losses associated with these perishable goods. To mitigate such losses, low-temperature storage (LTS) is widely implemented to reduce metabolic activity, delay senescence, and limit decay, thereby preserving quality [2]. However, LTS can also induce physiological damage in certain tropical and subtropical fruit and vegetables. Commodities such as citrus, bananas, avocados, and zucchinis are particularly susceptible to chilling injury (CI) when exposed to prolonged periods of low, non-freezing temperatures [3]. Common symptoms of CI in citrus fruit include rind browning, pitting, softening, and increased susceptibility to decay. Global losses attributed to CI are likely to be substantial. Approximately 50 % of globally traded postharvest commodities are sensitive to CI, with up to 33 % of these products lost annually [4].

In citrus, CI can be induced when fruit are exposed to prolonged storage or shipping below cultivar-specific threshold temperatures [4]. The disorder is not visibly expressed on the rind during cold storage and only becomes externally visible after transfer to shelf-life conditions, approximately 25 °C for 4 to 7 days [5]. Susceptibility to CI has been linked to multiple factors, including fruit species and cultivar, preharvest growing conditions, harvesting and transport practices, mechanical handling, postharvest and packaging processing, and the use of chemical treatments such as waxing or thiabendazole [[6], [7], [8]]. Industry experience further indicates that CI susceptibility is influenced by cooling conditions as air movement across the individual fruit rinds results in convective heat and mass transfer, which can accelerate cooling and moisture loss rates.

**Fig. 1:**
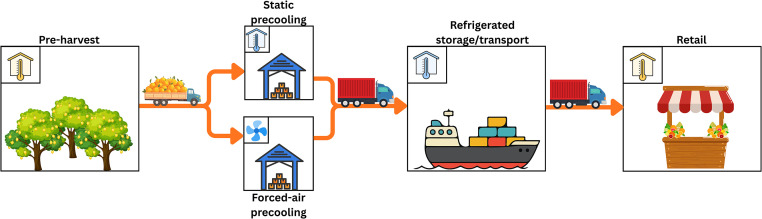
Process-flow schematic of the commercial citrus export supply chain, which serves as a representative example for most fresh produce. Orange arrows indicate product movement through the supply chain. Temperature icons denote environmental conditions: yellow houses represent ambient conditions, blue houses indicate cold storage, and the blue fan depicts forced-air cooling (FAC). Transport from the orchard to the precooling stage (FAC or static) is non-refrigerated, whereas the subsequent cold chain, from precooling through land and sea transport to retail, remains continuously refrigerated.

Research has demonstrated that both high- and low-temperature conditioning of certain fruit and vegetables, applied before long-term cold storage, can significantly reduce the incidence of CI [[9], [10], [11], [12]]. This indicates that the early phases of the postharvest cold chain, particularly the cold store period before shipping (Fig. 1), can play an important role in determining CI susceptibility. Appropriate precooling protocols, therefore, represent an important potential mitigation strategy. Precooling is defined as the rapid removal of field heat before long-term storage. Precooling effectiveness is determined mainly by the cooling rate, which is primarily a function of airflow produced by the fan configuration in the room and the delivery air temperature. Other factors impacting cooling rate include fruit size, packaging configuration, set-point temperature, and the thermal properties of the fruit, which vary with sugar content and the temperature of companion fruit.

The impact of precooling protocols on the susceptibility of CI has not been well investigated in the literature. This is largely because of the high variability and multiple interacting factors that influence the induction of the disorder. Current studies often excluded critical cold-chain phases, omitting either the precooling process or post-cooling storage and market simulation, which limits comparability and commercial relevance [[20], [21], [22]]. Furthermore, laboratory-scale forced-air precooling studies have focused on package design, heat transfer, or energy use, offering limited control of airspeed at individual fruit level [[13], [14], [15], [16], [17], [18], [19], [20]]. Trials were often run sequentially on different fruit batches, increasing variability. No prior work has systematically linked specific airspeed exposure and cooling-rate to fruit response. For example, high local airflow near vents may increase chilling injury [16], but conventional setups cannot isolate these effects. Controlled, uniform airflow exposure across many fruit will enable clearer analysis.

This work introduces a comprehensive, controlled approach using multiple precooling wind tunnel setups that precisely regulates airflow and temperature, enabling systematic evaluation of how different precooling protocols influence chilling injury across the entire cold chain.

The specific objectives of this work are to (i) characterise commercial precooling airflow and temperature conditions as device boundary conditions. (ii) Illustrate the design for a laboratory precooling wind tunnel simulator. (iii) Develop a chilling injury evaluation technique using the respective simulator and considering the citrus export supply chain from precooling to retail (Fig. 1).

## Method details

### Characterisation of commercial precooling conditions

The laboratory precooling wind tunnel simulator must be designed to operate within similar ranges to those found in commercial precooling operations. This entails experimentally determining air speeds during forced-air cooling (FAC) and measuring temperature cooling profiles during both FAC and room (static) cooling.

#### Cooling rate characterisation

**Fig. 2:**
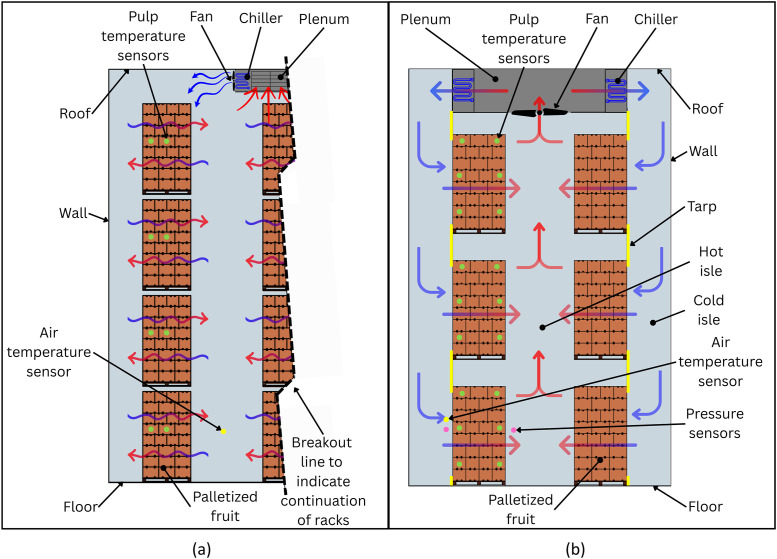
Simplified front-view schematic of a commercial precooling room. (a) Static room cooling: a ceiling-mounted refrigeration unit cools the bulk room air and pallets cool by natural convection. (b) Forced-air cooling: refrigerated air is discharged to the side aisles and drawn through the pallet stacks into a central plenum, then returned to the refrigeration unit. Green circles denote temperature-sensor positions. Blue arrows indicate cold-air supply and red arrows indicate warmer return flow.

To determine the spatiotemporal distribution of thermal exposure and response during precooling, fruit pulp and ambient temperatures were measured at a commercial facility. Thermistor probes (PB-5002–1M5) connected to Tinytag data loggers (TGP-4510 PLUS 2; Gemini Data Loggers, Cape Town 8001, South Africa) measured pulp and air temperatures in fruit. Sensor placement and precooling dynamics are shown in Fig. 2.

#### Estimating superficial inflow air speeds

The superficial air speed through the pallets was calculated from the pressure drop measured across multiple commercial FAC systems. Static pressure was recorded with a pressure transducer (A2G-25 air2guide, Wika, Lawrenceville, GA 30,043, USA). The airflow momentum loss coefficient for many of these pallet configurations are known, enabling its estimation [21]. The superficial air speeds were then determined from the measured pressure differential (ΔP) across a pallet (Fig. 2b), the produce depth relative to the flow direction (l), and the directional airflow resistance coefficient (ω), adapted from a previous study [22]:

(1) $$\bar{u}=\sqrt{\frac{\Delta P}{l\omega }}.$$

### Laboratory-scale testing

#### Equipment design

**Fig. 3:**
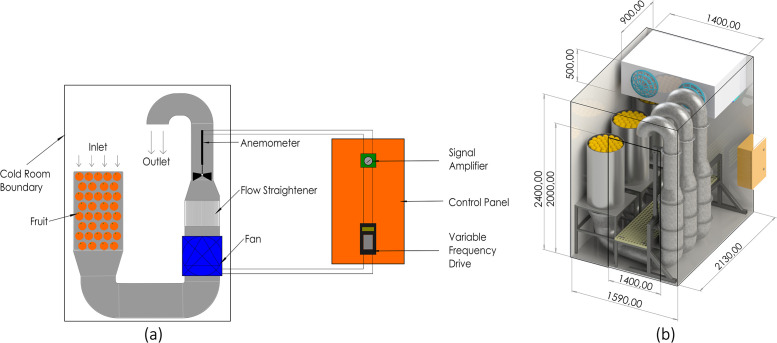
(a) Schematic of a FAC tunnel providing overview of the system operation, including airflow direction, instrumentation, and system components. (b) CAD rendering of three FAC tunnels installed within a cold room. The cold room's overall dimensions are indicated; the front and right-hand walls, door, and roof are hidden for clarity but outlined for spatial reference. The refrigeration unit (featuring two blue fan covers) is ceiling-mounted at the rear of the room. A yellow fibreglass walkway spans the ducting from the entrance to provide access to the test sections, which are loaded with citrus fruit to represent a typical trial configuration. Both the ducting and test sections are supported by mild steel frames resting on the cold room floor. The orange control box - containing the variable frequency drive and signal amplifier, is mounted externally to the right-hand wall.

A laboratory-scale facility was needed to control thermofluidic conditions. The design requirements for this facility included controllable air temperature, controllable airflow, and the ability to match and manipulate cooling rates observed during the commercial characterisation. Our approach was to design a convective cooling tunnel placed inside an existing cold room. The facility, a laboratory-scale forced-air cooling (LFAC) system, was developed to apply controlled air speeds to experimental fruit (Fig. 3a). Six LFAC tunnels were placed in two rooms (three per room; Fig. 3b), utilizing the rooms' independent refrigeration systems for applying prespecified delivery air temperature.

**Fig. 4:**
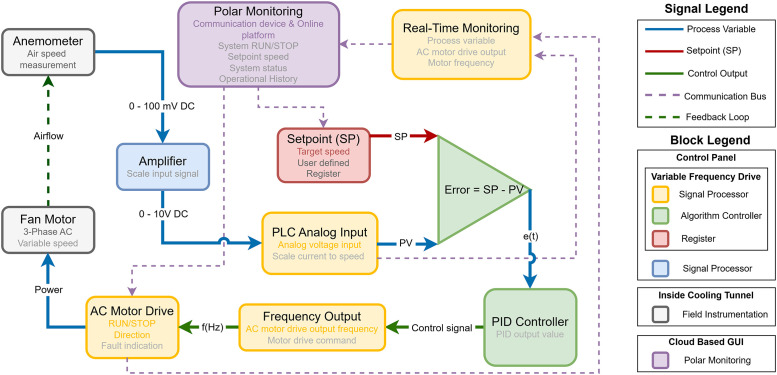
Communication and control schematic for the cooling tunnel system.

The closed-loop software framework (Fig. 4) operates on a variable frequency drive (VFD; Table 1) with integrated programmable logic controller (PLC) functionality. It is designed to regulate the fan motor speed based on real-time airflow measurements. The air speed is measured with a propeller anemometer, which generates a low-level DC voltage (0–100 mV). The anemometer output is amplified to a 0–10 V signal and scaled by the PLC's analogue input to a speed (m s^-1^) variable. The target speed setpoint is configured remotely through a cloud-based supervisory control and data acquisition (SCADA) platform (Polar Monitoring, Sandton, 2090, South Africa) via standard industrial RS-485 Modbus communication protocol. The control algorithm calculates the operational airspeed deviation from the set point and processes it through a PID (Proportional-Integral-Derivative) controller, which generates a frequency command proportional to the required speed correction. This command is converted to an AC output frequency (0–100 Hz) that drives the three-phase fan motor at variable speeds. The PLC continuously monitors motor drive parameters including fan motor frequency, superficial air speed, and fault conditions, transmitting this operational data back to the Polar Monitoring platform for alarm notification, and data logging.

**Table 1:** Cooling tunnel instrumentation.

Instrument	Model	Manufacturer
Inline axial flow fan	AP0314AP10/0.4	A.M.S Supplies (Pty) Ltd., Cape Town, South Africa
Self-powered gill propeller anemometer	27106T	R M Young Co, Michigan, United States of America
Weak signal amplifier module	LM358	Texas Instruments, Texas, United States of America
Variable frequency drive	Delta MS300	Delta Electronics, Taipei, China

**Fig. 5:**
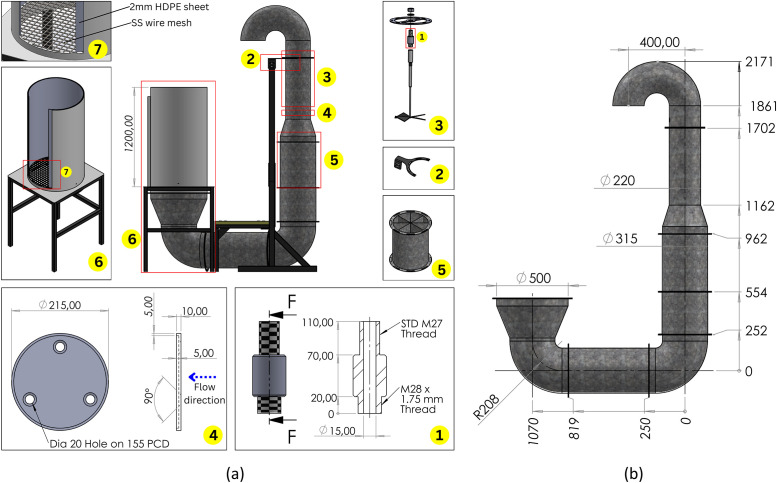
(a) Cooling tunnel with overall dimensions and specialised parts shown. Parts indicated: (1) Anemometer mount. (2) Anemometer assembly and exhaust duct stay. (3) Exploded view of anemometer mount assembly. (4) Orifice plate with detailed dimensions and flow direction indicated. (5) Star-type flow stabiliser. (6) Test section with cut-away to show internal parts. (7) Close-up view of test section bottom end. (b) Ducting assembly with general dimensions.

The test section (Fig. 5a) and subsequent ducting (Fig. 5b) was designed with a circular cross-section to promote axisymmetric flow, minimising secondary flows and simplifying the airflow speed profile to enable more controlled airflow conditions. Important design details included maximising transition piece lengths to limit flow disturbances and installing a star-type flow straightener to reduce swirl. The duct diameter was reduced at the anemometer inflow to minimise bypass, and two of the three tunnels were equipped with orifice plates to increase local flow airflow speeds above the anemometer's detection threshold. Exhaust air was directed downward to prevent recirculation.

The tunnel test section was designed with a height of 1.2 m to represent the horizontal airflow path through a full pallet of fruit in a commercial FAC setting, while allowing the packing density to be accurately replicated. To limit conductive heat transfer, direct fruit-metal contact was limited by installing a 2 mm high density polyethylene (HDPE) liner, which maintained a 10 mm annular air gap between the liner and the metal cylinder (Fig. 5a).

#### Equipment calibration

The LFAC tunnels were individually calibrated to address any bias flows at the anemometer section. The calibration was done by fitting the cooling tunnel exhaust with an inline flow rate measuring device. This enabled the recording of flow rates at quasi-steady-state intervals across the tunnel’s operational range. The flow rate was used to calculate the superficial airflow speed in the test section. A custom-built venturi with a pressure transducer (A2G-25 air2guide, Wika, Lawrenceville GA 30,043, USA) was utilised. The venturi was designed and calibrated by Chung [21] so that the relationship of the flow rate in the venturi was determined to scale according to the pressure differential between the inlet and the contraction section. In parallel, the output signal from the anemometer was recorded. The collected data was used to develop a regression function for each tunnel, with the resulting gradient (m) and offset (c) implemented as scaling parameters in the VFD control logic.

All sensors (e.g., A2G-25 air2guide, PB-5002–1M5 thermistor probes, and TGP-4510 PLUS 2 Tinytag data loggers) are periodically returned to their respective manufacturers for calibration, following the recommended service intervals. In addition, all equipment calibration was tested prior to experimentation to verify calibration accuracy [23]. The thermal sensors were tested using the Ice-Point bath method [24]. The pressure transducer was calibrated to a Standard calibration procedure outlined in the EURAMET cg-17 standard [25] using a Betz micromanometer as reference.

#### Uncertainty analysis of test equipment

An uncertainty analysis was conducted to quantify the system's performance and ensure non-overlapping operational ranges for specific parameters. Two primary sources of uncertainty were considered: the accuracy of the airflow calibration method and the system’s inherent control behaviour under experimental conditions. Each tunnel was individually assessed to capture variations in airflow speed performance across its operational range. The system was operated at a constant airflow speed for 4 h per set point, with measurements recorded at 5-minute intervals. The confidence interval (*Z*) was calculated using a stability metric [26]:

(2) $$Z=\frac{\sigma_{std}\sqrt{n}}{\sigma_{max}},$$

where $\sigma_{std}$ is the standard deviation, $\sigma_{max}$ is the maximum deviation from the setpoint, and n the number of sampling intervals. Total uncertainty ($\sigma_{total}$) was computed using the root-sum-square method [26], combining the system variation and calibration uncertainty ($\sigma_{calibration}$):

(3) $$\sigma_{total}=\sqrt{{\left(\sigma_{std}\right)}^{2}+{\left(\sigma_{calibration}\right)}^{2}\;}.$$

#### Fruit simulators

Fruit simulators were employed to reduce the amount of actual fruit required during trials. The simulators were Polypropylene plastic spheres (80 mm outside diameter) with a 0.3 mm wall thickness, filled with a saline solution (40 g L⁻¹ NaCl). The saline solution serves to minimise the growth of bacteria and limit the risk of contamination in the event of leakage.

#### Trial setup

This section outlines the methodology for operating the laboratory facility. In pre-trial preparations, healthy citrus fruit were randomly selected to minimise bias in CI sensitivity, and no postharvest treatments were applied to isolate the effects of the precooling protocols. Three replications of the trial were conducted to account for potential error.

**Fig. 6:**
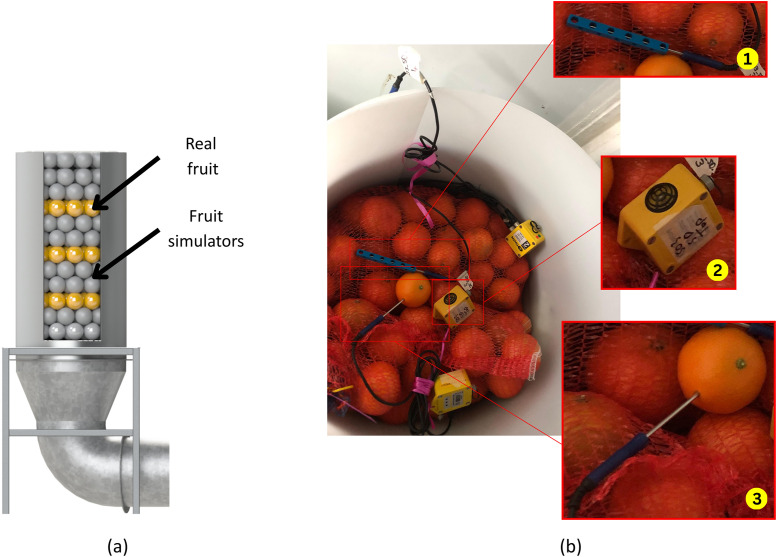
Trial preparation: (a) Fruit test section illustration with cut-away to show real-to-simulated fruit arrangement. (b) Photos showing sensor placement for every fruit level in a cooling tunnel during trial preparation, highlighting the following: (1) Thermistor probe with isolation cover fitted. (2) RH sensor placement. (3) Pulp temperature probe placement.

*Trial preparations*. Real and simulated fruit are divided into experimental units of 30 items, packed in 10 kg red knitted bags (Sakpro, White River, 1240, South Africa). The fruit bags are tagged and weighed. For each tunnel, 21 bags are prepared, six with citrus and fifteen with simulated fruit. The real to simulated fruit arrangement is illustrated in Fig. 6a.

Temperature monitoring is performed on each fruit level using PB-5002–1M5 thermistor probes connected to TGP-4510 PLUS 2 Tinytag data loggers (Gemini Data Loggers, Cape Town 8001, South Africa). Pulp temperatures were measured in selected fruit (fruit damaged by probe insertion were excluded from further analysis), and ambient temperature tracked using probes housed in custom 3D-printed (PLA) shields to minimise conductive interference. Fig. 6 shows the placement of fruit, spheres, and sensors within the tunnel.

*Precooling.* Two precooling treatments were applied simultaneously to the same batch of fruit. In the first cold room, air temperature was maintained at −0.6 °C for 72 h; in the second cold room, air temperature was held at 4.0 °C for 24 h, then reduced to −0.6 °C for 48 h. In both rooms, the wind tunnel system delivered continuous target air speeds of 0.05, 0.20, and 0.35 m s⁻¹ within the respective cooling tunnels. Each room also included an additional no-airflow control (0 m s⁻¹) treatment comprising an equal number of fruit stacked as a column of cartons and insulated equivalently to the tunnel packs, to isolate the effect of airflow.

*Low-temperature storage and market simulation.* After the 72-h precooling period, all probes were removed from the fruit. The trial fruit was transferred to a cold storage facility to simulate shipping conditions. Here, the temperature was maintained at −0.6 °C for 30 days. Following this, the fruit were moved to an ambient (unrefrigerated) room for 7 days to simulate a shelf-life. Ambient temperature and relative humidity were not actively controlled during this period, ranging from 18 to 28 °C and 41–83 %, respectively. These warmer conditions increase respiration rates and allow the rind disorders to be expressed.

*Post-trial analysis.* Immediately after the 7-day market simulation period, each fruit bag was reweighed using a digital scale to determine mass loss and the fruit were individually evaluated for chilling injury.

### Chilling injury evaluation

**Fig. 7:**
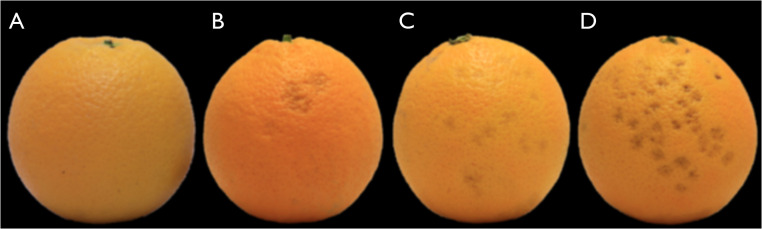
Citrus fruit with varying degrees of chilling injury.

Source: [28]

Commercially, markets have a zero tolerance for CI, and fruit with rind disorders are sorted on arrival at significant expense. The severity and proportion thus directly determine the economic and food losses. In alignment with commercial practice, the experimental fruit were visually inspected by a trained technician using standardised assessment metrics to estimate CI severity ($CI_{severity})$. It was scored on a standard four-level scale: (i) 0 = no damage (Fig. 7A), with no signs of chilling injury (no pitting), (ii) 1 = minor pitting of the rind (Fig. 7B), (iii) 2 = moderate pitting of the rind (Fig. 7C), and (iv) 3 = severe chilling injury symptoms (Fig. 7D), affecting >30 % of the rind [28].

To estimate the proportion of CI affected fruit ($CI_{induced}$), the formula:

(4) $$CI_{induced}=\frac{N_{affected}}{N_{evaluated}}*100,$$

was used. $N_{evaluated}$ is the total number of fruit evaluated, and $N_{affected}$ is the total amount of fruit with minor or more severe pitting [27]. To further investigate the symptom severity, the CI index ($CI_{index}$) was calculated as:

(5) $$CI_{index}=\sum_{i}CI_{severity,i}\left(0\;to\;3\right)*\frac{N_{score,i}}{N_{evaluated}},$$

where $N_{score,i}$ is the number of fruit in each score class.

## Method validation

### Characterisation of commercial precooling conditions

#### Cooling rates

Citrus pulp cooling rates were measured at a commercial citrus cold storage facility in KwaZulu-Natal, which was visited in September 2024. The facility housed dedicated FAC and static cooling rooms that are representative of typical commercial precooling facilities for citrus.

**Fig. 8:**
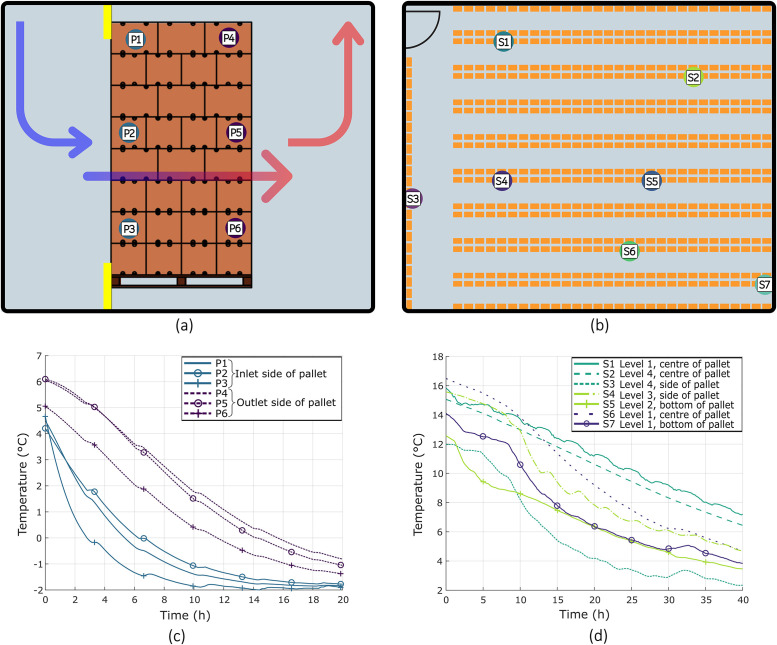
Temporal pulp temperature profiles of fruit measured at a commercial facility. (a) Cross-sectional view of a second-level pallet showing the experimental setup during forced-air cooling investigation. Arrows indicate airflow direction from the inlet, through the pallet, to the exhaust side returning to the chiller (as shown in Fig. 2). Yellow shading denotes tarps used to prevent bypass flow. Sensor positions (P1–P6) are marked, with corresponding temperature profiles shown in panel c. (b) Top-view schematic of the static cold room layout depicting rows of racked pallets (orange rectangles). Sensor positions (S1–S7) are indicated, with corresponding temperature profiles shown in panel d. (c) Pulp temperature profiles recorded during FAC. Sensors P1–P3 represent inlet-side positions, while P4–P6 represent outlet-side positions. (d) Pulp temperature profiles recorded during static cooling, showing spatial temperature variation across different pallet levels and positions within the cold room.

The FAC room accommodates up to 60 fully loaded pallets, 20 pallets on each level. The room measured approximately 11 m in height, 6 m in width, and 11 m in depth. During the investigation, the facility housed 20 pallets, all on the second level. Each pallet consisted of 90 full telescopic cartons (A15C-S2), each carton packed with approximately 16.5 kg of Valencia oranges. A single stacked pallet was probed on multiple levels on the inlet and outlet side cartons, as indicated in Fig. 8a. The set point temperature of the chiller during the FAC cycle was set to −1.5 °C.

The static cooling room, with a floor area of 25.6 m by 35.3 m, has a total capacity of 2080 pallets racked on four vertical levels. During the investigation, the facility housed approximately 1000 pallets. The Valencia oranges were also packed in A15C-S2 cartons. Temperature probes were inserted into fruit positioned in the bottom, centre and side locations and placed randomly in the room (Fig. 8b). The room temperature was maintained at 0 °C. These measurements aimed to characterise the minimum (static; Fig. 8d) and maximum (FAC; Fig. 8c) pulp cooling rates. These results served as benchmarks for validating the laboratory precooling setup.

#### Estimating superficial inflow velocities

Data was collected at four South African FAC facilities to characterise operational pressure drop ranges. The measured pressure drop was applied to the airflow resistance coefficient, which is 771 kg m^-4^ for horizontal flow through an A15C-S2 carton pallet stack [21]. The resulting pressure drops and calculated superficial air velocities (Eqn. (1)) across and through the pallets are listed in Table 2.

**Table 2:** Pressure drop and calculated superficial inlet velocities for each facility.

	Facility #1	Facility #2	Facility #3	Facility #4
Static pressure (Pa)	17	28	32	67
Superficial airflow speed (m s^-1^)	0.14	0.17	0.19	0.27

The superficial air velocities represent the bulk flow conditions and do not account for the local variations in air velocity between individual fruit, where tortuosity and packing density cause higher or lower local velocities. To account for this, the cooling tunnels were (i) intentionally packed densely to replicate commercial configurations and reflect realistic packing densities, and (ii) operated at approximately 30 % of the calculated lowest (Facility #1, Table 2) and 30 % above the highest (Facility #4, Table 2) superficial airflow speeds. This resulted in tunnel airflow targets of 0.05, 0.20, and 0.35 m s⁻¹. These velocities are specific to citrus in A15C-S2 cartons, similar pressure–airflow characterisation would thus be required for other packaging/fruit combinations.

### System characterisation

#### Calibration

**Fig. 9:**
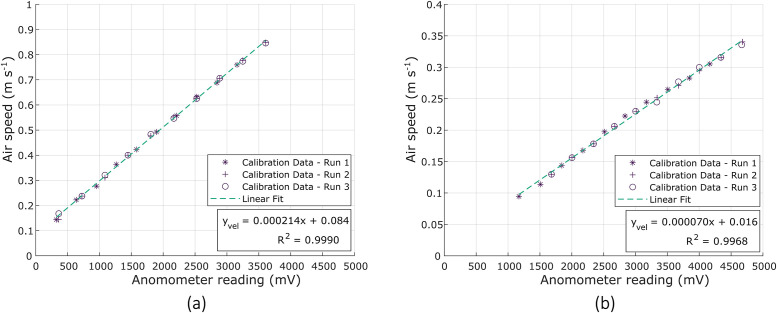
Calibration curve comparing the superficial airflow speed in the test section of the tunnel to the anemometer output reading with a linear regression curve fitted, and coefficient of determination indicated. Graph (a) is the tunnel suited for higher velocities, while (b) represents the lower airflow speed setup where the orifice plate was fitted to assist the velocity cut-in threshold of the anemometer.

Calibration was performed by recording the anemometer output current across a range of airspeeds for three replicates. The resulting linear regression function was used to interpret anemometer readings and regulate the system, as detailed in Section 2.2.2. The results (Fig. 9) from the repeated tests showed a good correlation (*R* > 0.99) to a linear fit.

#### Uncertainty analysis

**Fig. 10:**
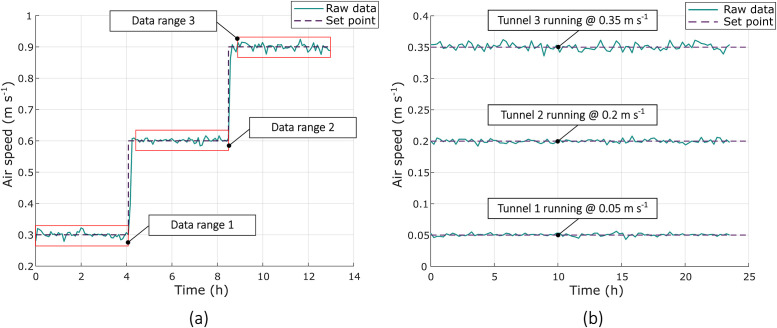
Airflow speed performance data used to characterise airflow control in the cooling tunnel system. (a) Airflow speed stability of a single tunnel operated at three target setpoints spanning its operational range. Red boxes indicate the time windows used for the uncertainty analysis discussed above. (b) Comparison of airflow speed control across three tunnels, each operating at its intended setpoint, demonstrating the system’s ability to deliver differentiable airflow treatments.

An uncertainty analysis was required because the system’s interlinked components can accumulate error. This shows that airflow variability remained low enough for the treatment speeds to remain clearly distinct. Fig. 10a illustrates a fully loaded LFAC tunnel’s performance at three target velocities, 0.30 m s^-1^, 0.60 m s^-1^, and 0.90 m s^-1^. Standard deviations were 0.01 m s^-1^, 0.06 m s^-1^, and 0.01 m s^-1^, respectively, with corresponding maximum deviations from the setpoint of 0.022 m s^-1^, 0.016 m s^-1^, and 0.024 m s^-1^. For all three air speed setpoints, Z (calculated with Eqn. (2)) exceeded the 2.75 threshold for a 99 % confidence level, indicating statistically stable airflow conditions [26]. These yielded total uncertainties (Eqn. (3)) of 0.30 ± 0.01 m s^-1^, 0.60 ± 0.02 m s^-1^, and 0.90 ± 0.02 m s^-1^, corresponding to relative uncertainties of 3.6 %, 2.3 %, and 2.3 %, respectively. The results confirm that the airflow remains stable and well-characterised across the operational range of the machine.

Fig. 10b shows a comparison of all three fully loaded tunnels operating at a trial-realistic setpoint. This confirms that tunnel airspeed variation allows the application of differentiable treatments. Together, these results confirm the system’s airflow control capabilities and confirm that the tunnels operate within well-characterised, statistically robust uncertainty margins suitable for comparative trials.

Note that fruit cooling rates are a factor of multiple factors including air speed, fruit size/properties and air temperature. Fruit pulp temperatures were thus measured directly. Uncertainty for the pulp temperature values was assumed from the manufacturer specifications (± 0.35 °C within the temperature range of the trials).

#### Cooling rate validation

**Fig. 11:**
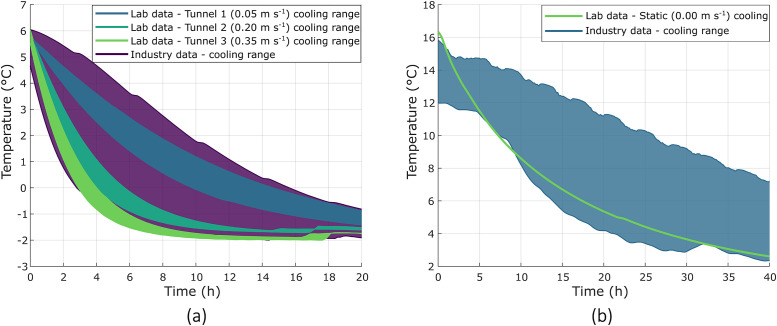
Overlay of laboratory and commercial cooling data for validation of the laboratory setup. Shaded regions represent the range between the fastest and slowest cooling responses. (a) Comparison of LFAC cooling tunnels with a commercial FAC cycle. (b) Comparison of laboratory static cooling with cooling ranges recorded during commercial static cooling.

As a final confirmation that the system is performing as expected, the systems cooling performance was benchmarked against commercial precooling data. The LFAC reproduced the commercial ranges of air temperature and pulp temperature cooling rates (Fig. 11), confirming that the system emulates commercial conditions.

### Chilling injury induction validation

**Fig. 12:**
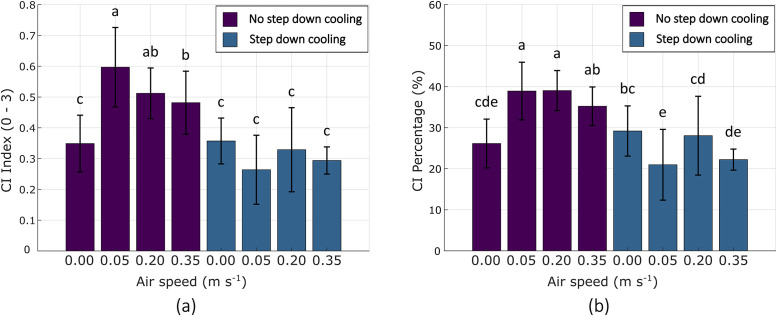
Bar graph reporting CI severity for citrus precooling trials expressed as (a) chilling injury index, and (b) chilling injury percentage.

A step-down and no-step cooling protocol trial was evaluated using the LFAC setup. These preliminary results are used to validate the method's capacity to induce chilling injury in citrus fruit through the application of varying air speeds and cooling rates. Fig. 12 presents CI results from this citrus trial, showing differentiable responses under controlled airflow and ambient temperature conditions. A one-way ANOVA (α = 0.05) confirmed statistically significant differences between treatments, demonstrating the system’s operational effectiveness and validity for CI research. While additional trials are required to establish broader trends, these early results illustrate the type of information that can be generated using the developed methodology to evaluate CI as a function of different precooling parameters.

## Limitations

*Commercial validation benchmark representativeness*. The commercial facility used for validation operated at partial capacity during measurements. The FAC room, with a total capacity of 60 pallets, housed only 20 pallets (approximately 33 % capacity) during data collection. This loading density influences cooling rates as there is less thermal load for the existing cooling capacity.

It was necessary to run a reduced load to minimise interference with ongoing operations. The fruit were removed after the trial to collect data loggers and then precooled again, placing them at an increased risk of chilling injury. Consequently, the trial size was reduced. However, FAC tunnels are frequently fractionally loaded in industry, so this configuration remains representative of commercial practice. Although it is not representative of a fully loaded system, the validation data provide a baseline for system testing.

Once benchmarked against these commercial conditions, the laboratory setup will be used to investigate both slower and faster cooling ranges. The laboratory system’s adjustable airflow speeds enable a broader range of precooling conditions to be systematically evaluated beyond those observed during the validation measurements.

*Scale* vs. *industrial complexity*. While the laboratory setup replicates industrial temperature profiles, it cannot fully capture the heterogeneity and complexities of airflow distribution in large-scale commercial pallet stacks and room layouts. Especially considering the practical limitation of physically measuring every fruit's pulp temperature during commercial precooling. For a deeper understanding, a computational fluid dynamics model of a stacked pallet can be developed to better understand the conditions.

*Thermal behaviour of material*. The use of simulated fruit may not fully represent the thermal properties, surface characteristics, or metabolic responses of real fruit, potentially affecting the accuracy of heat transfer dynamics during trials and should be avoided, if possible.

## Supplementary material *and/or* additional information [OPTIONAL]

None

## Ethics statements

None required

## CRediT authorship contribution statement

**Eugene Sadie:** Methodology, Software, Validation, Formal analysis, Investigation, Data curation, Writing – original draft, Visualization. **Corne Coetzee:** Writing – review & editing, Supervision. **Mike Owen:** Writing – review & editing, Supervision. **Marli Kleyn:** Investigation, Formal analysis, Writing – review & editing. **Tarl Berry:** Conceptualization, Resources, Writing – review & editing, Supervision, Funding acquisition.

## Declaration of competing interest

The authors declare that they have no known competing financial interests or personal relationships that could have appeared to influence the work reported in this paper.

## Data Availability

Data will be made available on request.
